# Awareness of cervical cancer and willingness to be vaccinated against human papillomavirus in Mozambican adolescent girls

**DOI:** 10.1016/j.pvr.2018.04.004

**Published:** 2018-04-14

**Authors:** Azucena Bardají, Carolina Mindu, Orvalho J. Augusto, Aina Casellas, Olga Cambaco, Egas Simbine, Graça Matsinhe, Eusébio Macete, Clara Menéndez, Esperança Sevene, Khátia Munguambe

**Affiliations:** a ISGlobal, Hospital Clínic - Universitat de Barcelona, Barcelona, Spain; b Consorcio de Investigación Biomédica en Red de Epidemiología y Salud Pública (CIBERESP), Madrid, Spain; c Centro de Investigação em Saúde de Manhiça (CISM), Rua 12, Cambeve CP 1929, Maputo, Mozambique; d Aga Khan Foundation, Mozambique; e Mozambique Ministry of Health. Avenida Eduardo Mondlane, Maputo, Mozambique; f Faculdade de Medicina, Universidade Eduardo Mondlane, AV. Salvador Allende, 702 Maputo, Mozambique

**Keywords:** Human papillomavirus, Vaccine, Cervical cancer, Mozambique, Awareness and acceptability

## Abstract

Sub-Saharan Africa concentrates the largest burden of cervical cancer worldwide. The introduction of the HPV vaccination in this region is urgent and strategic to meet global health targets. This was a cross-sectional study conducted in Mozambique prior to the first round of the HPV vaccine demonstration programme. It targeted girls aged 10–19 years old identified from schools and households. Face-to-face structured interviews were conducted. A total of 1147 adolescents were enrolled in three selected districts of the country. Most girls [84% (967/1147)] had heard of cervical cancer, while 76% believed that cervical cancer could be prevented. However only 33% (373/1144) of girls recognized having ever heard of HPV. When girls were asked whether they would accept to be vaccinated if a vaccine was available in Mozambique, 91% (1025/1130) answered positively. Girls from the HPV demonstration districts showed higher awareness on HPV and cervical cancer, and willingness to be vaccinated. This study anticipates high acceptability of the HPV vaccine in Mozambique and high awareness about cervical cancer, despite low HPV knowledge. These results highlight that targeted health education programmes are critical for acceptance of new tools, and are encouraging for the reduction of cervical cancer related mortality and morbidity in Mozambique.

## Highlights

• Cervical cancer incidence rates in Mozambique are among the highest in the world.
• Introduction of HPV vaccination in Mozambique is an urgent and necessary strategy to meet global targets on women´s health.
• The awareness on cervical cancer, and anticipated acceptance of HPV vaccination was assessed in Mozambican adolescent girls.
• The study anticipates high acceptability of HPV vaccine and adequate awareness about cervical cancer.
• These results are timely in the context towards the implementation of the HPV vaccine at a national scale in Mozambique.

## 1 Introduction

Cervical cancer is the fourth most common cancer among women worldwide, affecting half a million women each year [1]. Around 85% of the cases occur in developing countries [2]. It is the leading cause of cancer mortality in women in many countries, being responsible for 270,000 deaths every year [1]. Sub-Saharan Africa concentrates the largest burden of cervical cancer worldwide, where this condition is the leading cause of cancer mortality among women [2]. This is largely due to the fact that in this region, the prevalence of human papillomavirus (HPV) infection is the highest in the world, that cervical cancer screening programmes are rare or operate with limitations, and that high-quality treatment is either unavailable or unaffordable [3]. The highly prevalent co-infection with human immunodeficiency virus (HIV) in these settings also increases the risk of HPV infection and progression to cancer [4], [5].

Cervical cancer incidence rates in Mozambique are among the highest in the world, standing at 44.8/100,000 women per year (estimates for 2012). It is the most frequent cancer among women of all ages and the leading cause of female cancer-related mortality countrywide [6], [7]. Data available in the country on HPV infection and cervical cancer is limited. The studies that have been conducted in Mozambique revealed the presence of HPV-DNA in up to 40% of asymptomatic women of reproductive age [8], [9], and detected HPV in all invasive cervical cancer cases, being genotypes 16 and 18 the most commonly found, accounting for up to 80% of cervical cancer cases [9], [10], [11], [12]. In addition, the national estimates on HIV prevalence are among the highest in sub-Saharan Africa with 15% (95%CI 13.9–16) of individuals aged 15–49 years old being HIV-infected, with higher prevalence among women compared to men [13]. In Mozambique, a cervical cancer screening programme, based on the visual inspection with acetic acid (VIA) technique, was launched in 2009 in four provinces, and is gradually rolling out to the rest of the country [14]. Despite its recognized advantages, the limited proportion of health facilities in Mozambique currently implementing the program [15], the fact that the screening program only reaches those women who seek care at health facilities, the low acceptability of immediate treatment among confirmed positive cases [15], high turn-over of staff, and the technique´s sub-optimal specificity and sensitivity [12], [14], jeopardise its success as an effective approach to reduce cervical cancer incidence country-wide.

It is clear that the introduction of the HPV vaccination to prevent cervical cancer in low and middle-income countries is an urgent and strategic approach to meet global health targets on women´s health [16]. In 2011, the Global Alliance for Vaccination and Immunization (GAVI) formalized its commitment to support the introduction of the HPV vaccine in priority low-income countries. Under this initiative, in 2013, Mozambique was granted GAVI support for a two-year demonstration programme, targeting adolescent girls, in preparation for a national scale up of vaccination against HPV. In Mozambique no data existed to suggest the best approach to target this age group in a feasible way. The evaluation of the feasibility of HPV vaccination among adolescent girls in contexts such as that of Mozambique is critical for the success and sustainability of HPV vaccine introduction before moving towards national-scale implementation.

Political barriers and health systems weakness are pointed out as major limitations for the success and the sustainability of HPV vaccination programmes [3]. It is recognized that as vaccines become available in low-income countries, the understanding of the factors interfering with and/or contributing to the success of HPV vaccination programmes becomes a priority [17], [18]. It is critical to assess beliefs, awareness, attitudes, and behaviours of the populations and providers involved, conduct acceptability studies and evaluate operational aspects related to vaccine delivery, to better understand potential barriers and facilitators to effective vaccine implementation [19]. Most acceptability studies of HPV vaccination have been conducted in high-income countries and usually indicate a high interest in vaccines. However, safety concerns, cost and socio-cultural factors have been frequently identified as barriers, potentially interfering with uptake [20], [21]. Studies thus far conducted in sub-Saharan Africa (SSA) report high acceptance of HPV vaccination, although cervical cancer awareness and knowledge is often poor [19], [20], [21] and safety concerns mainly based on misunderstandings regarding vaccine´s damage to adolescents´ fertility [18] and perceived unsafe practices in administrating the vaccine are consistently reported [22], [23], [24], [25], [26], [27], [28], [29], [30], [31], [32], [33], [34], [35]. In Mozambique, studies exploring the acceptability of new vaccines and preventive strategies, suggest a good receptiveness at the community level [36], [37], [38], [39]. However, little is known on the awareness and knowledge about the diseases being prevented by such vaccines, including HPV infection and cervical cancer [15].

This study aimed to assess the awareness on cervical cancer and HPV infection, and anticipated acceptance of HPV vaccination for cervical cancer prevention among adolescent girls in three districts of Mozambique in the framework of an HPV vaccine demonstration programme. Since HPV vaccine strategies prioritize girls prior to sexual debut, it was also an objective to understand the pattern for initiation of sexual practices, perceived risk and self-reported protection against cervical cancer among Mozambican adolescent girls. This information will serve to understand the barriers and facilitators for effective HPV vaccine implementation, and it may be of relevance to other countries in sub-Saharan Africa and elsewhere with similar health problems and limited resources.

## 2 Materials and methods

### 2.1 Study areas and participants

The study targeted adolescent girls aged 10–19 years old. This group was selected based on the study´s intention to document wider issues about prevention of HPV, as a sexually transmitted infection among adolescents [40], but also to capture specific issues related to HPV vaccination among the foreseen vaccination target group for Mozambique.

The study was conducted between August 2013 and May 2014 in three geographically different areas of Mozambique, namely Ka-Mavota (peri-urban, Maputo city) and Manhiça (semi-rural, 80 km north Maputo city) districts, both in Maputo province, in Southern Mozambique, and in Mocímboa da Praia district (rural), in Cabo Delgado province, in Northern Mozambique (see Fig. 1) [13], [18].

### 2.2 Study design and data collection

This was a quantitative, cross-sectional study conducted prior to the first round of the HPV vaccination campaign in the context of the HPV vaccine demonstration programme in Mozambique, and concomitantly to the education and communication activities that preceded the HPV vaccination pilot programme. Two of the three districts where this study was conducted (Mocímboa da Praia and Manhiça), were target districts for the demonstration programme. Eligible participants were identified and selected randomly from schools and households within the selected study areas and invited to participate in the study. The reason for this mixed method of recruitment was to ensure that girls not enrolled in school were also captured. To capture this reality, a conservative assumption was made that 2/3 of the girls would be enrolled in school. In each district, 10 schools were randomly selected and served as the sampling frame. In each school, 30 girls were randomly selected and invited to participate in the study. In the community, the target sample was of at least 100 girls randomly selected per district, which were captured from 10 randomly selected neighbourhoods per district and identified either via the demographic surveillance platform (in Manhiça) or a list of known 10-year old girls by the neighbourhood secretaries.

A total sample size of 1248 adolescent girls, with a minimum sample of 410–420 girls per site, was foreseen in order to fulfil the study objectives, based on a sample size calculation to detect 50% acceptance to vaccinate against HPV (with 90% power and allowing for 10% refusals). Each participant was provided with a study information sheet written in the language in which the respondent was most comfortable (either Portuguese or the main local language of the respective District). Most interviews were conducted in Portuguese and very few of them in the local language.

A paper-based standardised questionnaire was administered to adolescent girls by trained female interviewers with experience conducting community-based questionnaires, assessing awareness on cervical cancer and HPV infection, perception of their own risks with regards to HPV transmission, acceptability and willingness of adolescent girls to be vaccinated against HPV, and perceived risks and benefits of HPV vaccination. For the assessment of sexual behaviour patterns and perception of risk of cervical cancer, due to the nature of the questions, only girls aged 15–19 were inquired.

### 2.3 Ethics statement

Ethical approval for this study was granted by the Institutional Review Board of the Manhiça Health Research Centre (CIBS-CISM) [Reg. No. CIBS_CISM_09/13] and by the Ethics Review Committee of the Hospital Clinic of Barcelona, Spain (CEIC) [Reg. No. HCB/2013/0903]. The study was conducted in accordance with the Good Clinical Practice Guidelines set up by the WHO, and under the provisions of the Declaration of Helsinki, and local rules and regulations. Adolescent girls were enrolled after they gave written consent (if 18 years of age or older) and assent accompanied by parents´ or guardians´ written informed consent (if younger than 18 years of age).

### 2.4 Data management and statistical analysis

A standardised system for data entry, data management, and statistical analysis was established. All data were double entered by two independent data clerks into form-specific databases (REDCap) [41]. Validation and cleaning were done using the same software. Data was analysed using Stata 14.1 (Stata Corporation, College Station, TX, USA). Data analysis consisted of descriptive statistics including frequency distribution for demographic variables and responses obtained in relation to awareness on cervical cancer and HPV infection, sexual behaviour patterns and perception of health risks, preferred venues for vaccine delivery and reasons for acceptance of HPV vaccine. Willingness of adolescent girls to be vaccinated was recorded in terms of the proportion of adolescent girls that hypothetically would accept to be vaccinated. Data are presented as mean (SD) for continuous variables. Differences between proportions were compared using the Pearson´s *chi-squared* test or Fisher's exact test if the application conditions of the former were not met. For quantitative variables, the ANOVA test was used for group comparisons. Associations between outcome variables (knowledge of HPV and cervical cancer, vaccine acceptability and sexual behaviour) and socio-demographic variables (study site, age group, level of education, marital status, literacy, and occupation) were explored using logistic regression. Multivariable models were built using a stepwise procedure, where the variables study site and age category were forced to remain in the model. The significance level was set at p < 0.05. Missing values were coded as such and excluded from analysis. Adolescents were defined, according to WHO definition, as young people between the ages of 10 and 19 years [40]. Level of education was categorised as having completed primary school studies or studies higher than primary school. Two age groups were defined, whether being between 10 and 14 years old, or between 15 and 19 years old.

## 3 Results

### 3.1 Demographic characteristics of study adolescent girls

A total of 1147 Mozambican adolescent girls from three selected districts (357 in Manhiça, 402 in Ka-Mavota, and 388 in Mocímboa da Praia), accepted to participate in the study, of which 85% (970/1141) were recruited at school and 15% (171/1141) at their households. Mean age of participants was 14.4 years [standard deviation (SD) 2.8], being 627 girls between 10 and 14 years of age, and 520 girls between 15 and 19 years of age. Baseline characteristics of study participants are shown in Table 1.

**Table 1:** Demographic characteristics of adolescent girls.

				**Study site**						
		**Total**		**Mocímboa da Praia**		**Manhiça**		**Ka-Mavota**		
		**N**		**N**	**(%)**	**N**	**(%)**	**N**	**(%)**	**p-value**^a^
**Participants**		1147		388	(34)	357	(31)	402	(35)	
**Age** (years)*		14.4	(2.8)	13.4	(2.4)	14.0	(2.5)	15.7	(2.9)	<0.001
		**n**	**(%)**	**n**	**(%)**	**n**	**(%)**	**n**	**(%)**	
**Age group (years)**	10–14	627	(55)	265	(68)	215	(60)	147	(37)	<0.001
	15–19	520	(45)	123	(32)	142	(40)	255	(63)	
		**n/N**	**(%)**	**n/N**	**(%)**	**n/N**	**(%)**	**n/N**	**(%)**	
**Site of interview**	At school	970/1141	(85)	327/388	(84)	309/356	(87)	334/397	(84)	0.523
	At the community	171/1141	(15)	61/388	(16)	47/356	(13)	63/397	(16)	
**Level of education**	Primary school	690/1094	(63)	302/356	(85)	249/357	(70)	139/381	(36)	<0.001
	Higher than primary	404/1094	(37)	54/356	(15)	108/357	(30)	242/381	(64)	
**Literacy**	Reads and/or writes	986/1125	(88)	256/373	(69)	336/354	(95)	394/398	(99)	<0.001
**Marital status**	Single	1071/1142	(94)	355/387	(92)	322/356	(90)	394/399	(99)	<0.001
	Joined or married	71/1142	(6)	32/387	(8)	34/356	(10)	5/399	(1)	
**Occupation**	Non-worker	944/1128	(84)	308/385	(80)	286/350	(82)	350/393	(89)	0.001
	Worker	184/1128	(16)	77/385	(20)	64/350	(18)	43/393	(11)	

a Chi-squared test.

* Arithmetic Mean (SD), p-value with ANOVA test.

### 3.2 Awareness of HPV infection and cervical cancer

The majority, [84% (967/1147)] of girls had heard of cervical cancer, and 64% of them (725/1139) reported the uterus as the main organ affected by cervical cancer; however, only 33% of the participants (373/1144) recognized having ever heard of HPV infection; with higher knowledge on HPV in the districts where the HPV demonstration programme was being conducted (58% in Mocímboa da Praia, and 28% in Manhiça) Seventy-one percent of adolescent girls (685/963) considered sexual transmission as the principal mode of acquisition of cervical cancer followed by history of cancer in the family (21%) (See Table 2). Regarding sources of information about HPV and cervical cancer, the National HPV vaccine demonstration programme, which was under preparation at the time of the conduction of the present study.

**Table 2:** Knowledge and awareness of HPV infection and cervical cancer (CC) among adolescent girls.

				**Study site**						
		**Total**		**Mocímboa da Praia**		**Manhiça**		**Ka-Mavota**		
		**n/N**	**(%)**	**n/N**	**(%)**	**n/N**	**(%)**	**n/N**	**(%)**	p-value*
**Ever heard of cervical cancer?**		967/1147	(84)	296/388	(76)	336/357	(94)	335/402	(83)	<0.001
**Organs affected by CC**	Uterus	725/1139	(64)	226/385	(59)	283/356	(79)	216/398	(54)	<0.001
	Breast	183/1139	(16)	10/385	(3)	46/356	(13)	127/398	(32)	
	Others	51/1139	(4)	44/385	(11)	7/356	(2)	0/398	(0)	
	Don´t know	180/1139	(16)	105/385	(27)	20/356	(6)	55/398	(14)	
**Recognized signs of CC**	Abnormal vaginal bleeding	344/960	(36)	167/292	(57)	114/336	(34)	63/332	(19)	<0.001
	Vaginal discharge	331/960	(34)	143/292	(49)	134/336	(40)	54/332	(16)	<0.001
	Dyspareunia	281/960	(29)	39/292	(13)	103/336	(31)	139/332	(42)	<0.001
**Mode of acquisition of CC**	Sexual transmission	685/963	(71)	243/294	(83)	249/336	(74)	193/333	(58)	<0.001
	History of cancer in the family	199/963	(21)	36/294	(12)	60/336	(18)	103/333	(31)	
	Others	9/963	(1)	2/294	(1)	5/336	(1)	2/333	(1)	
	Don´t know	70/963	(7)	13/294	(4)	22/336	(7)	35/333	(11)	
**Ever heard of HPV infection?**	Yes	373/1144	(33)	224/387	(58)	98/356	(28)	51/401	(13)	<0.001
**Source of information**	Through HPV Demo Program	256/608	(42)	157/237	(66)	58/100	(58)	41/271	(15)	<0.001
**for HPV and CC**	Mass media	203/608	(33)	37/237	(16)	7/100	(7)	159/271	(59)	<0.001
	At the health centre	76/608	(13)	20/237	(8)	11/100	(11)	45/271	(17)	0.019
	At the community	44/608	(7)	11/237	(5)	6/100	(6)	27/271	(10)	0.061

* Chi-squared test.

### 3.3 Knowledge on prevention of cervical cancer and HPV vaccine

Seventy six per cent (866/1146) of study participants were aware that cervical cancer can be prevented (see Table 3). When asked about which tools or strategies could prevent cervical cancer, 64% (551/855) reported vaccination, though only 36% (407/1133) had ever heard of a specific vaccine to prevent it (see Table 3, Table 4). About best timing when the vaccine should be given, 62% (532/865) of participants reported that before sexual debut, however 15% (133/865) reported that at any age.

**Table 3:** Knowledge and awareness about prevention of cervical cancer (CC) and HPV vaccine among adolescent girls.

				**Study site**						
		**Total**		**Mocímboa da Praia**		**Manhiça**		**Ka-Mavota**		
		**n/N**	**(%)**	**n/N**	**(%)**	**n/N**	**(%)**	**n/N**	**(%)**	p-value*
**Thinks cervical cancer**	Yes	866/1146	(76)	269/388	(69)	322/357	(90)	275/401	(69)	<0.001
**can be prevented**	No	93/1146	(8)	22/388	(6)	23/357	(6)	48/401	(12)	
	Don´t know	187/1146	(16)	97/388	(25)	12/357	(3)	78/401	(19)	
**If yes, modes of prevention**	Vaccination	551/855	(64)	144/268	(54)	224/319	(70)	183/268	(68)	<0.001
	By using condoms	151/855	(18)	47/268	(18)	65/319	(20)	39/268	(15)	0.183
	Delaying sexual initiation	145/855	(17)	80/268	(30)	37/319	(12)	28/268	(10)	<0.001
	Less sexual partners	104/855	(12)	46/268	(17)	34/319	(11)	24/268	(9)	0.009
**When you think vaccination**	Before sexual initiation	532/865	(62)	176/260	(68)	213/329	(65)	143/276	(52)	<0.001
**should be given**	At any age	133/865	(15)	40/260	(15)	24/329	(7)	69/276	(25)	
	After 18 years of age	102/865	(12)	18/260	(7)	35/329	(11)	49/276	(18)	
	After sexual inititiation	72/865	(8)	14/260	(5)	52/329	(16)	6/276	(2)	
	Don´t know	17/865	(2)	5/260	(2)	4/329	(1)	8/276	(3)	
	Others	9/865	(1)	7/260	(3)	1/329	(0)	1/276	(0)	

* Chi-squared test.

**Table 4:** Acceptability and willingness to vaccinate against HPV among adolescent girls.

				**Study site**						
		**Total**		**Mocímboa da Praia**		**Manhiça**		**Ka-Mavota**		
		**N**	**(%)**	**n**	**(%)**	**n**	**(%)**	**n**	**(%)**	p-value*
**If available in Mozambique, would accept**		1025/1130	(91)	349/387	(90)	318/353	(90)	358/390	(92)	0.658
**to be vaccinated?**										
**Preferred place to be**	At the health centre	620/1012	(61)	162/345	(47)	193/314	(61)	265/353	(75)	<0.001
**Vaccinated**	At school	347/1012	(34)	75/345	(51)	111/314	(35)	1/353	(17)	<0.001
	At the community	164/1012	(16)	77/345	(22)	53/314	(17)	34/353	(10)	<0.001

* Chi-squared test.

### 3.4 Acceptability of HPV vaccine by Mozambican adolescent girls

When girls were asked whether they would accept to be vaccinated if a vaccine was available in Mozambique, 91% (1025/1130) of girls answered positively. This high HPV vaccine acceptability was observed across all study sites (see Table 4). Across sites, the preferred place to be vaccinated was the health centre (61%), followed by the school (34%) and the community (16%).

### 3.5 Factors associated with awareness about HPV and cervical cancer and acceptability of HPV vaccine among Mozambican adolescent girls

Adolescent girls from Mocímboa da Praia, in Northern Mozambique, showed higher knowledge about what HPV infection and cervical cancer represent and its prevention, followed by girls from Manhiça district, in Southern Mozambique (see Table 5). A better understanding of HPV infection and its relation to cervical cancer was seen among older adolescents (15–19 years of age), compared to young ones (10–14 years). The level of education (higher than primary) was associated with a greater knowledge about cervical cancer and how it could be prevented.

**Table 5:** Factors associated with knowledge about HPV, cervical cancer and its prevention: adjusted multivariate model.

		HPV knowledge*				Cervical cancer knowledge*				Knowledge about prevention of cervical cancer*			
		**n/N**	**OR**^a^	**(95% CI)**	**p-value**	**n/N**	**OR**	**(95% CI)**	**p-value**	**n/N**	**OR**	**(95% CI)**	**p-value**
**Study site**	Mocímboa d/Praia	223/386	1		**<0.001**	272/356	1		**<0.001**	246/265	1		**<0.001**
	Manhiça	98/355	0.25	(0.18–0.34)		336/357	4.49	(2.70–7.47)		322/345	0.99	(0.53–1.87)	
	Kha-Mavota	51/398	0.09	(0.06–0.13)		319/381	0.98	(0.65–1.48)		262/309	0.28	(0.15–0.51)	
**Age group**	10–14	195/622	1		**<0.001**	480/602	1		**0.003**	434/488	1		0.586
(years)	15–19	177/517	1.79	(1.31–2.44)		447/492	1.94	(1.25–3.03)		396/431	1.17	(0.66–2.06)	
**Marital status**	Single	326/1068	1		**0.002**								
	Joined/married	46/71	2.50	(1.41–4.44)									
**Education**	Primary school					557/690	1		**0.007**	496/558	1		**0.008**
	> than primary					370/404	2.02	(1.21–3.37)		334/361	2.37	(1.26–4.48)	

a OR: Odds Ratio.

* Number of observations: 1139 in HPV knowledge model, 1094 in cervical cancer knowledge model and 919 in prevention model.

No differences were seen on the acceptability of the HPV vaccine across study sites (p = 0.118) or age groups (p = 0.323) (see Supplementary Table S1). Factors associated with increased acceptability of HPV vaccine were level of education (adjusted OR, 4.85, [95% CI 2.24–10.53]; p < 0.001 for girls with education higher than primary school compare to those with only primary school studies), having some knowledge about cervical cancer (adjusted OR, 2.29, [95% CI 1.36–3.83]; p = 0.002), and having some knowledge about prevention of cervical cancer (adjusted OR, 2.62, [95% CI 1.40–4.90]; p = 0.007).

### 3.6 Pattern of sexual behaviour and perception of risk of cervical cancer among older adolescent girls (15–19 years of age)

Overall, 94% (481/514) of older adolescent girls interviewed had heard of sexually transmitted infections (STIs). Of them, 84% (405/480) reported that STIs could be prevented by using condoms, and 23% (109/480) by having only one sexual partner (see Table 6). Sixty one percent of them (314/514) said to be sexually active by the time of the interview, being the mean reported age at first sexual intercourse of 15.7 years [standard deviation (SD) = 1.6]. Twenty three per cent (70/310) of the participants reported early debut of sexual relations (before 15 years of age), being Mocímboa da Praia the district where higher proportion of early beginners (55%) was observed (see Table 6).

**Table 6:** Pattern of sexual behaviour and perception of risk against cervical cancer (adolescent girls ≥15 years).

				**Study site**							
		**Total**		**Mocímboa da Praia**		**Manhiça**			**Kha-Mavota**		
		**N**	**(%)**	**n**	**(%)**	**n**	**(%)**		**n**	**(%)**	p-value*
**Ever heard of sexually transmitted infections?**		481/514	(94)	108/120	(90)	134/141	(95)		239/253	(94)	0.184
**If yes, how can be prevented**	Using condoms	405/480	(84)	67/107	(63)	122/134	(91)		216/239	(90)	<0.001
	Having only 1 partner	109/480	(23)	54/107	(50)	20/134	(15)		35/239	(15)	<0.001
**Have initiated sexual relations**		314/514	(61)	89/120	(74)	69/141	(49)		156/253	(62)	<0.001
**Age at first sexual intercouse** (years)¶		15.7 (1.6) [310]		14.4 (1.6) [86]		15.6 (1.7) [68]			16.4 (1.2) 156]		<0.001
**Early initiation of sexual relations** (age <15 y)		70/310	(23)	47/86	(55)	14/68		(21)	9/156	(6)	<0.001
**Number of sexual partners so far**	1	132/307	(43)	33/86	(38)	39/69		(57)	60/152	(39)	0.012
	2	121/307	(39)	30/86	(35)	24/69		(35)	67/152	(44)	
	3 or more	54/307	(18)	23/86	(27)	6/69		(9)	25/152	(16)	
**Use of condoms**		237/313	(76)	29/88	(33)	61/69		(88)	147/156	(94)	<0.001
**If yes, how often you use them?**	Always	123/236	(52)	9/29	(31)	24/61		(39)	90/146	(62)	<0.001
	Sometimes	113/236	(48)	20/29	(69)	37/61		(61)	56/146	(38)	
**Feels at risk of cervical cancer**		257/451	(57)	80/115	(70)	71/124		(57)	106/212	(50)	0.003

* Chi-squared test.

¶ Arithmetic Mean (SD) [n], p-value with ANOVA test.

Of those girls who had initiated sexual relations, 43% (132/307) reported one previous sexual partner, 39% (121/307) two sexual partners, and 18% (54/307) three or more sexual partners. Regarding use of condoms, 76% (237/313) reported to use them (52% always and 48% sometimes). Nearly 60% (257/451) of participants felt they were at risk of cervical cancer, being Mocímboa da Praia (70%) and Manhiça (57%) the districts where perception of such risk was higher.

## 4 Discussion

To our knowledge, this is the first study conducted in Mozambique assessing the awareness of cervical cancer and willingness to be vaccinated against HPV among adolescent girls. This study was carried out in advance of the roll out of HPV vaccination countrywide. Overall, knowledge about cervical cancer and its prevention was high among girls, with 84% of them having heard of cervical cancer, while 76% believed that cervical cancer could be prevented, and recognising vaccination as the main strategy to prevent it. However, the level of awareness about HPV infection was low, with only a third of girls that recognized having heard about this infection. In addition, acceptability levels of the HPV vaccine was high across sites, with an overall 91% of the girls willing to be vaccinated should the vaccine be available in the country.

Most studies in SSA on awareness about cervical cancer and HPV vaccine consistently reported that while levels of knowledge are generally low, willingness to vaccinate against HPV is high [17], [18], [19], [20], [21], [22], [23], [24], [25], [26], [27], [29], [30], [31], [32], [33], [35], [42], [43]. In this study, both awareness of cervical cancer (despite low awareness of HPV infection), and willingness to vaccinate against HPV was very high across the three study districts. Our findings on acceptability are in accordance with results from other settings in SSA and worldwide. In our population, main determinants of acceptability and willingness to be vaccinated against HPV were the level of education, together with previous knowledge about cervical cancer and available tools to prevent it.

Several studies in SSA have shown high levels of awareness in the population and acceptability of the HPV vaccine following HPV vaccination campaigns [18], [20], [25]. This study showed both high awareness and anticipated acceptability, particularly in the two study districts (Mocímboa da Praia and Manhiça) target for the first vaccination campaign of the HPV vaccine demonstration programme in Mozambique. This reflects the effect of already on-going circulating information (provided as part of education and communication activities), prior to vaccination campaign in pilot areas. Indeed, in these two districts the main source of information for HPV and cervical cancer reported by study participants were the information activities preceding the HPV vaccination campaign. These findings align with the content of the information spread through the demonstration awareness campaign, which consisted in community radio spots broadcasted in advance of the campaign, question and answers booklets distributed in the communities, and posters posted at health facilities and schools. While posters and radio spots focused on the vaccine and its relevance to a healthy present and future life, the questions and answers booklet focused on the female reproductive tract, characteristics of cervical cancer, modes of transmission and prevention.

Interestingly, the preferred place for vaccination was the health centre (61%) across sites and age groups, despite the limited routine contacts of adolescents with the health system and the generally low attendance to adolescent-specific services in settings like this [44]. This finding is not aligned, in principle, with the demonstration program strategy being considered by the Ministry of Health of Mozambique (MoH) at the time. It would be risky for the MoH to attempt a solely facility-based approach grounded on girls preference, given the existing evidence of low adherence to health facilities among healthy adolescent girls, and high coverage of school-based vaccination demonstrated in Rwanda, South Africa [45], Tanzania and Uganda [46]. In order to address girls preferences, the appropriate option to the Mozambique MoH would be to focus on school-based delivery as the main approach [17] in addition to a community- and health facility-based strategy to reach those girls who never enrolled in school or dropped out by the age they were to be vaccinated (10 years).

It is noteworthy to mention that while we found a perceived understanding by adolescent girls that sexually transmitted infections (STIs) were associated with increased risk of suffering from cervical cancer, there was a huge disparity between awareness of STIs in general and awareness of HPV infection (only 33% of adolescent girls had ever heard about HPV, versus 91% who were aware of other STIs, such as HIV or syphilis). In addition, while willingness to be vaccinated to prevent cervical cancer was high, participants showed little understanding about best timing to be vaccinated and the link between the efficacy of the vaccine and sexual debut [47]. The low awareness of HPV infection reported together with the high willingness to be vaccinated seemed paradoxical, but this might be explained by the adolescents´ understanding and perception that the purpose of vaccination was to prevent cervical cancer rather than to avoid a sexually transmitted infection.

Mean age at first sexual intercourse was of 15 years across districts and nearly 25% of girls had initiated sexual relations before the age of 15. This finding are aligned with the rates of child marriage in Mozambique, being the highest in Northern provinces; 17.6% of girls in Cabo Delgado between the ages of 20 and 24 years were married before 15 years of age [48]. These findings, including early initiation of sexual relations (before 15 years of age) and reported number of sexual partners, are similar to those from other settings in SSA [14], [49]. This is, coupled with the inconsistent pattern of condom use reported by the study participants, of particular concern, given the high prevalence of HIV infection in the country, also highly prevalent in the youngest populations, and the synergistic effects of the HIV and HPV co-infection [13], [50].

Despite the wide anticipated overall acceptance of the HPV vaccine observed across sites this study, we found differences on knowledge and perceived risk for cervical cancer. Adolescent girls from the district in the periphery of the capital, Ka-Mavota, showed the poorest knowledge about cervical cancer and the HPV vaccine. This may be explained because this area was not a target HPV vaccination demonstration site, reflecting the imbalance of information spread between targeted and non-targeted areas. When comparing the two targeted sites for HPV vaccine demonstration, baseline knowledge either on HPV and vaccines for prevention of cervical cancer was the highest in Mocímboa da Praia, a remote rural area in Northern Mozambique, compared to Manhiça, also a rural area, but relatively proximate to the capital city in the South. Moreover, in Mocímboa district a much higher proportion of adolescent girls reported feeling at risk of cervical cancer compared to the other sites in the south of the country. The results on higher awareness about HPV and cervical cancer among Mocímboa da Praia adolescents seem to contrast their reported riskier sexual behaviour, where the average age at sexual debut was lower compared to other districts [51], and the proportion of girls reporting early initiation of sexual relations (before 15 years) and those reporting having had 3 or more sexual partners the highest. Such contrast between knowledge, perception of risk and actual risk behaviours had been reported previously in Mozambique [49]. An explanation for this finding might be that their experience about early initiation of sexual and reproductive life [52], [53] leads to a higher receptiveness to the idea of a vaccine to prevent cervical cancer.

## 5 Conclusions

In conclusion, our study anticipated high acceptability of HPV vaccine in Mozambique indicated by willingness to be vaccinated among adolescents across different settings of the country, as well as the adequate awareness about cervical cancer, prior to the roll out of vaccination against HPV. Specific aspects of knowledge of aetiology of cervical cancer need improvement. Our results highlight that relevant targeted health education programmes across the country, as those provided as part of the HPV vaccine demonstration programme, are critical for the acceptance of new tools to prevent cervical cancer, and that delivery strategies should address socio-cultural specificities across the country. The results coming from this study are encouraging and favourable to advance towards reduction in cervical cancer related mortality and morbidity in Mozambique.

## Declaration of interests

We declare no competing interests.

## Funding

This work was supported by the Department for International Cooperation, Barcelona City Hall, Spain [Grant No. 12S05844]; the Aga Khan Foundation (AKF); and the Global Alliance for Vaccination and Immunization (GAVI) [Grant Numbers 1415-MOZ-19a-X, 1415-MOZ-24a-Y]. The sponsors of the study had no role in study design, data collection, data analysis, data interpretation, or writing of the report, except for the Agha Kahn Foundation, which participated in the study design and data collection. The study was coordinated by the Centro de Investigação em Saúde de Manhiça (CISM), Mozambique, in collaboration with the Universidade Eduardo Mondlane, Faculdade de Medicina, Maputo, Mozambique, and the Barcelona Institute for Global Health (ISGlobal)/Barcelona Centre for International Health Research, Hospital Clínic-Universitat de Barcelona, Spain.

## Contributors

KM, CaM and ES designed the study. KM, CaM, OC and ES organised investigations at study districts, implemented and supervised the study. CaM, OC and KM enrolled study participants and data collection. AB, AC, OA, KM were responsible for data management. AB and AC prepared the analytical plan and analysed data. AB wrote the manuscript. CaM, OA, OC, AC, ESi, GM, EM, ES, CM and KM commented on the manuscript. All authors contributed to discussion and interpretation of results and review and have seen and approved the final version of the manuscript.
