# Supplementary material for: Awareness of cervical cancer and willingness to be vaccinated against human papillomavirus in Mozambican adolescent girls
Source: Papillomavirus Res. 2018 Apr 14;5:156–62. doi: 10.1016/j.pvr.2018.04.004 (PMC6046684; doi:10.1016/j.pvr.2018.04.004)
Supplement: Supplementary Table S1 — Supplementary material [file mmc1.docx]

**Supplementary files**

Figure S1. Geographic location of study districts (Mozambique)


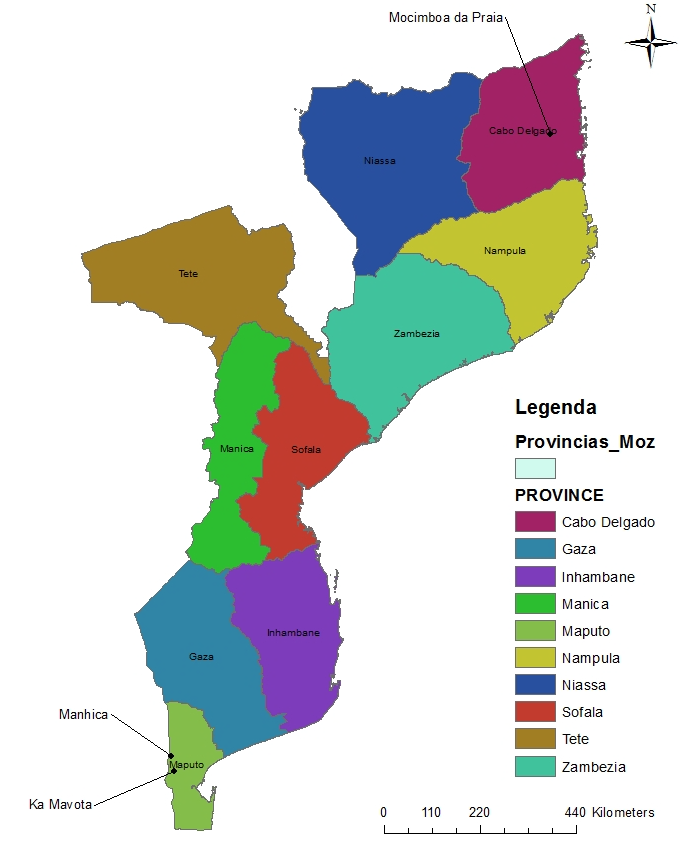


Table S1. Factors associated with HPV vaccine acceptability among adolescent girls: adjusted multivariate model

|  | |  | **Acceptability of HPV vaccine *** | | | | | | | | | |  |
| --- | --- | --- | --- | --- | --- | --- | --- | --- | --- | --- | --- | --- | --- |
|  | |  | **n/N** | | **OR** ¶ | | | **(95% CI)** | | **p-value** | | |  |
| **Study site** | Mocímboa da Praia | | 321/355 | | 1 | | |  | | 0.018 | | |  |
|  | Manhiça | | 318/353 | | 0.60 | | | (0.34-1.04) | |  | | |  |
|  | Kha-Mavota | | 338/369 | | 0.61 | | | (0.35-1.08) | |  | | |  |
| **Age group** (years) | 10-14 | | 515/591 | | 1 | | |  | | 0.323 | | |  |
|  | 15-19 | | 462/486 | | 1.31 | | | (0.76-2.26) | |  | | |  |
| **Level of education** | Primary school | | 590/680 | | 1 | | |  | | **<0.001** | | |  |
|  | Higher than primary school | | 387/397 | | 4.85 | | | (2.24-10.53) | |  | | |  |
| **Having some knowledge about CC** | No | | 129/162 | | 1 | | |  | | **0.002** | | |  |
|  | Yes | | 848/915 | | 2.29 | | | (1.36-3.83) | |  | | |  |
| **Having some knowledge about** | No | | 70/88 | | 1 | | |  | | **0.007** | | |  |
| **prevention of CC** | Yes | | 764/820 | | 2.62 | | | (1.40-4.90) | |  | | |  |
|  | Don´t know | | 143/169 | | 1.52 | | | (0.74-3.13) | |  | | |  |
| * Number of observations: 1077 | | | | | | | | | | | | | |
| ¶ OR: Odds Ratio | |  | |  | |  |  |  |  |  |  |  | |
